# Author Correction: Subcellular pathways through VGluT3-expressing mouse amacrine cells provide locally tuned object-motion-selective signals in the retina

**DOI:** 10.1038/s41467-024-48983-x

**Published:** 2024-06-05

**Authors:** Karl Friedrichsen, Jen-Chun Hsiang, Chin-I Lin, Liam McCoy, Katia Valkova, Daniel Kerschensteiner, Josh L. Morgan

**Affiliations:** 1 Department of Ophthalmology and Visual Sciences, Washington University in St. Louis, St. Louis, MO USA; 2 Department of Neuroscience, Washington University in St. Louis, St. Louis, MO USA; 3 Department of Biomedical Engineering, Washington University in St. Louis, St. Louis, MO USA; 4 Graduate Program in Neuroscience, Washington University in St. Louis, St. Louis, USA

**Keywords:** Neural circuits, Retina, 3-D reconstruction

Correction to: *Nature Communications* 10.1038/s41467-024-46996-0, published online 5 April 2024

The original version of this article incorrectly omitted Daniel Kerschensteiner as a corresponding author. This has been corrected in both the PDF and HTML versions of the Article.

