# Supplementary material for: Subcellular pathways through VGluT3-expressing mouse amacrine cells provide locally tuned object-motion-selective signals in the retina
Source: Nat Commun. 2024 Apr 5;15:2965. doi: 10.1038/s41467-024-46996-0 (PMC10997783; doi:10.1038/s41467-024-46996-0)
Supplement: Supplementary file 1 — Supplementary Information [file 41467_2024_46996_MOESM1_ESM.pdf]

Subcellular pathways through VGluT3-expressing mouse amacrine cells provide locally tuned object-motion-selective signals in the retina.

SUPPLEMENTARY INFORMATION

cell ID#	arbor µm	Convex Hull Area	Major Axis	Minor Axis	Orientation
2	1,632	3,061	61	41	87
3	1,526	3,750	85	42	88
4	1,350	3,557	61	53	73
5	807	4,996	89	52	79
13	1,223	4,457	86	50	49
14	918	3,026	68	40	-83

Supplementary Table 1: Table showing arbor length and shape for the six most reconstructed VG3s.

	number of bipolar cells	total number of synapses to	mean number of synapses	Synapses per pairing
bc3a	25	227	9.08 (1.303)	3.97 (0.401)
bc3b	9	31	3.44 (1.015)	1.77 (0.249)
bc4	15	35	2.33 (0.398)	1.87 (0.233)
bc5o	18	58	3.22 (0.527)	1.77 (0.157)
bc5i	11	32	2.91 (0.948)	1.44 (0.287)
bc5t	8	19	2.37 (0.532)	1.62 (0.288)
xbc	7	17	2.43 (0.481)	1.57 (0.207)
bc6	6	10	1.67 (0.211)	1.33 (0.164)

Supplementary Table 2. Cell and synapse numbers for types of bipolar cells found innervating the VG3 plexus. Last column refers to the number of synapses formed between each bipolar cell and each retinal ganglion cell.

	number of cells	total number of synapses from	Synapses per pairing (SEM)
25	1	1	1.00 (0.000)
28	1	5	1.67 (0.667)
2an	2	4	1.00 (0.000)
37	9	45	2.05 (0.326)
3i	1	3	1.50 (0.500)
4i	2	25	2.27 (0.359)
4on	3	21	1.91 (0.476)
4ow	3	55	3.67 (1.153)
5si	1	14	3.50 (1.658)
5so	1	1	1.00 (0.000)
5ti	6	66	3.67 (0.667)
5to	1	5	2.50 (1.500)
63	3	41	2.93 (0.633)
6sn	2	6	1.20 (0.200)
6sw	5	53	2.65 (0.437)
6t	1	3	1.50 (0.500)
7i	1	2	1.00 (0.000)
85	1	4	4.00 (0.000)
8w	2	2	1.00 (0.000)
m3	1	3	1.00 (0.000)

Supplementary Table 3. Cell and synapse number for each RGC type innervated by the VG3 plexus. Last column refers to the number of synapses formed between each VG3 cell and each retinal ganglion cell.

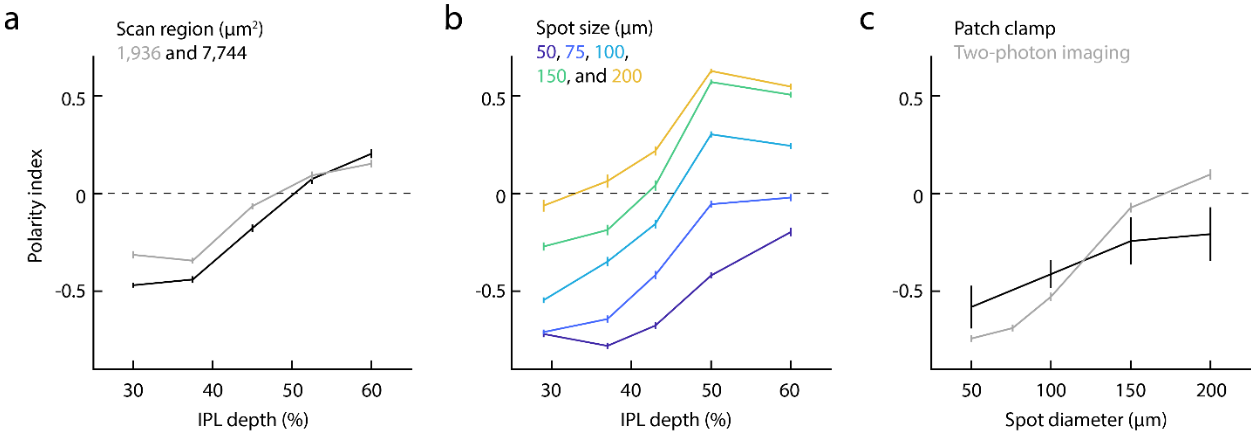

Supplementary Fig. 1: Stimulus size shapes response polarity. **a,** The size of scan region has little effect on how the ON/OFF calcium response polarity of VG3s changes with IPL depth (two mice). **b,** The polarity of VG3 responses shift from OFF biased towards ON biased as spot size increases (eight mice). **c,** The effect of stimulus size on response polarity can be seen at the soma by two-photon imaging (eight mice) and patch clamp recordings (28 mice).

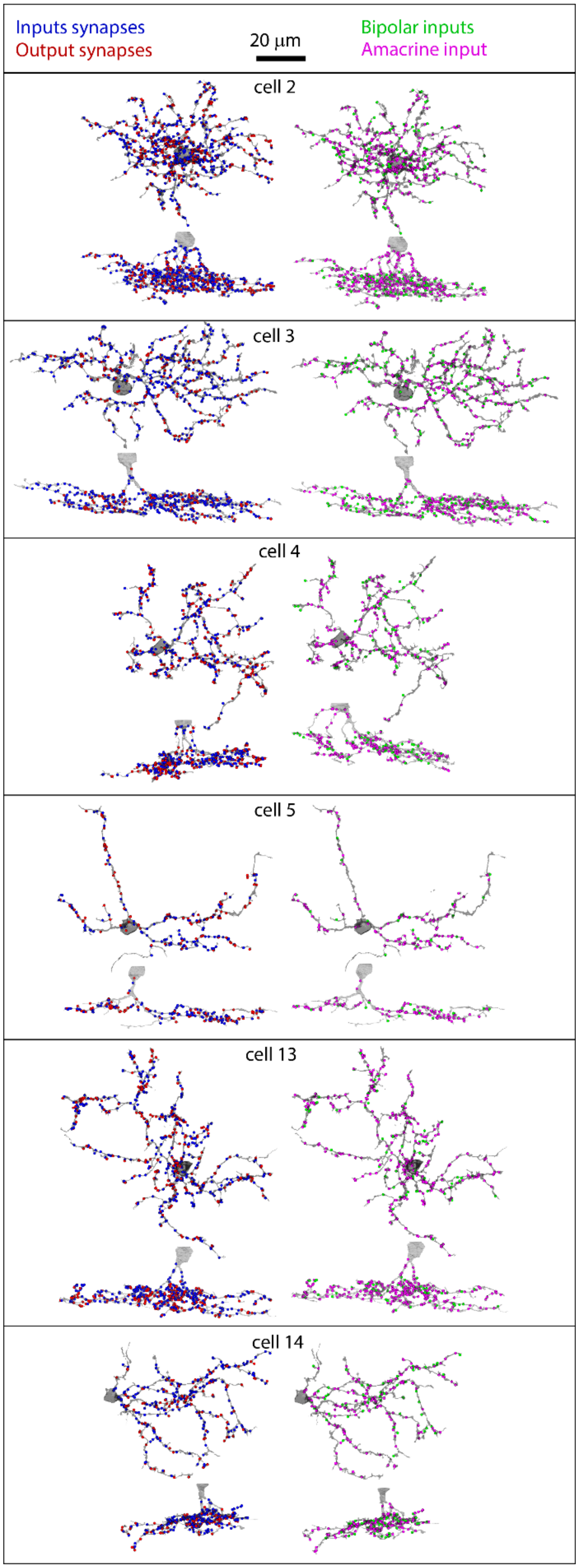

Supplementary Fig. 2: Synapse distributions for the six most reconstructed VG3s. Left shows input (blue) and output (red) synapses. Right shows bipolar cell (green) and amacrine cell (magenta) input synapses.

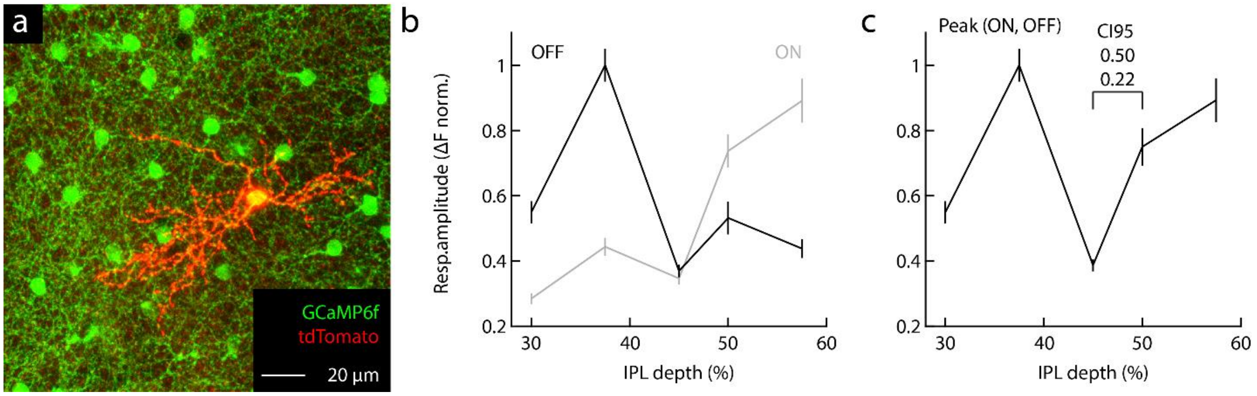

Supplementary Fig. 3: Calcium response amplitude dips in the center of the IPL. **a,** Representative image of an individual VG3 amacrine cell labeled with tdTomato by in vivo electroporation in a mouse in which all VG3 cells express GCaMP6f. **b,** Analysis of calcium response amplitudes (ΔF norm.) to ON and OFF stimuli (100 µm diameter spot) as a function of IPL depths. **c,** Analogous to (b) for the peak amplitudes across ON and OFF responses. The difference between the normalized response amplitude of the VG3 center (IPL depth 45%) and IPL depth 50% is 0.22 to 0.50 (CI95, n = 63, 65 rois).

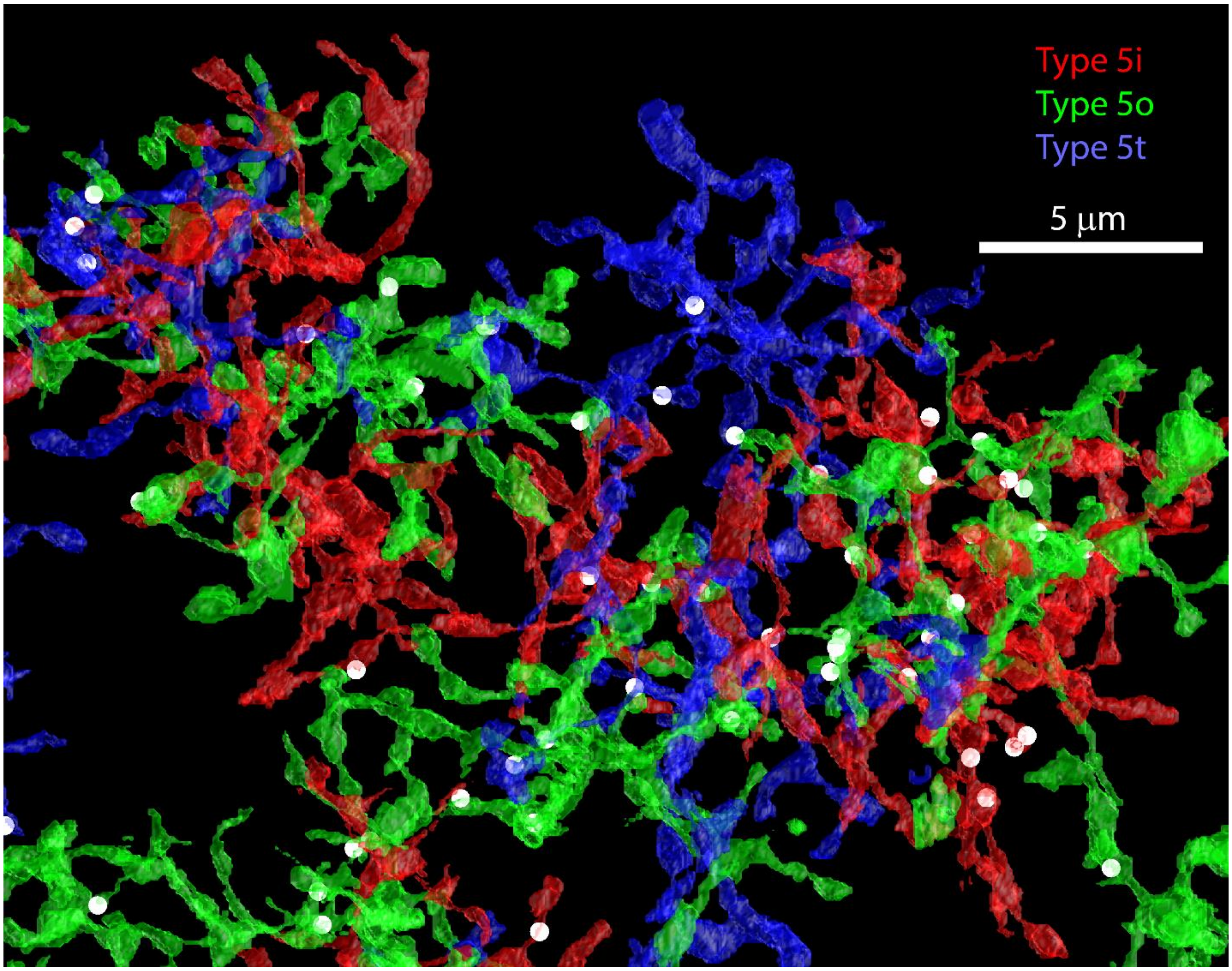

Supplementary Fig. 4: Top-down view of reconstructions of the three types of type 5 bipolar cells innervating the VG3 plexus. The position of synapses between the bipolar cells and VG3s are shown by white dots.

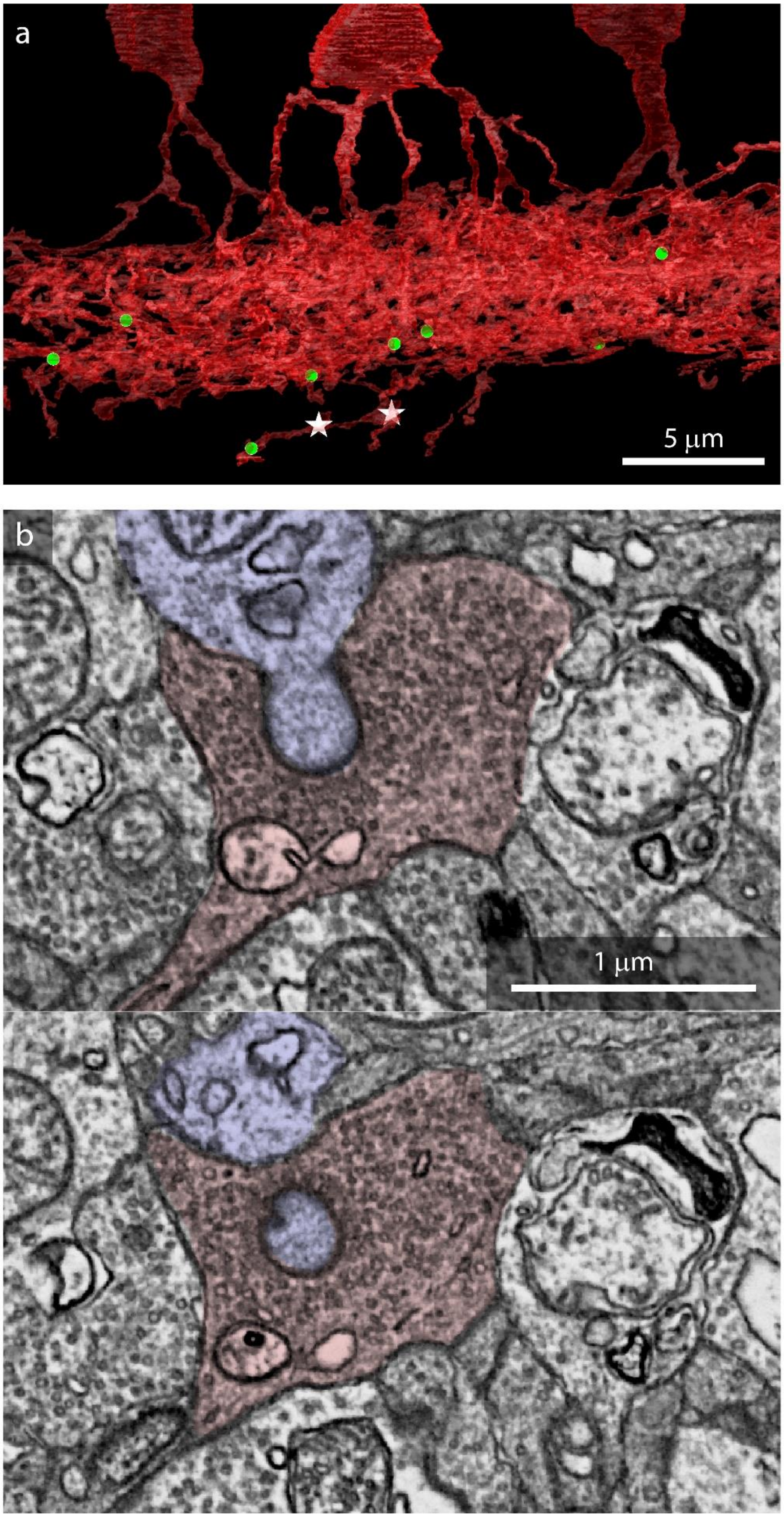

Supplementary Fig. 5: Synapses between VG3s and RGCs. **a,** One VG3 (red) process strays from the main plexus and forms two synapses (stars) on two type 8w (sustained ON) RGCs. The position of synapses between type 6 bipolar cells and the VG3 plexus are shown with green dots. **b,** Two sections through an exceptionally large synapse between a VG3 and a type 73 (suppressed by contrast) RGC. Note the vesicle rich active zone engulfing the RGC spine.

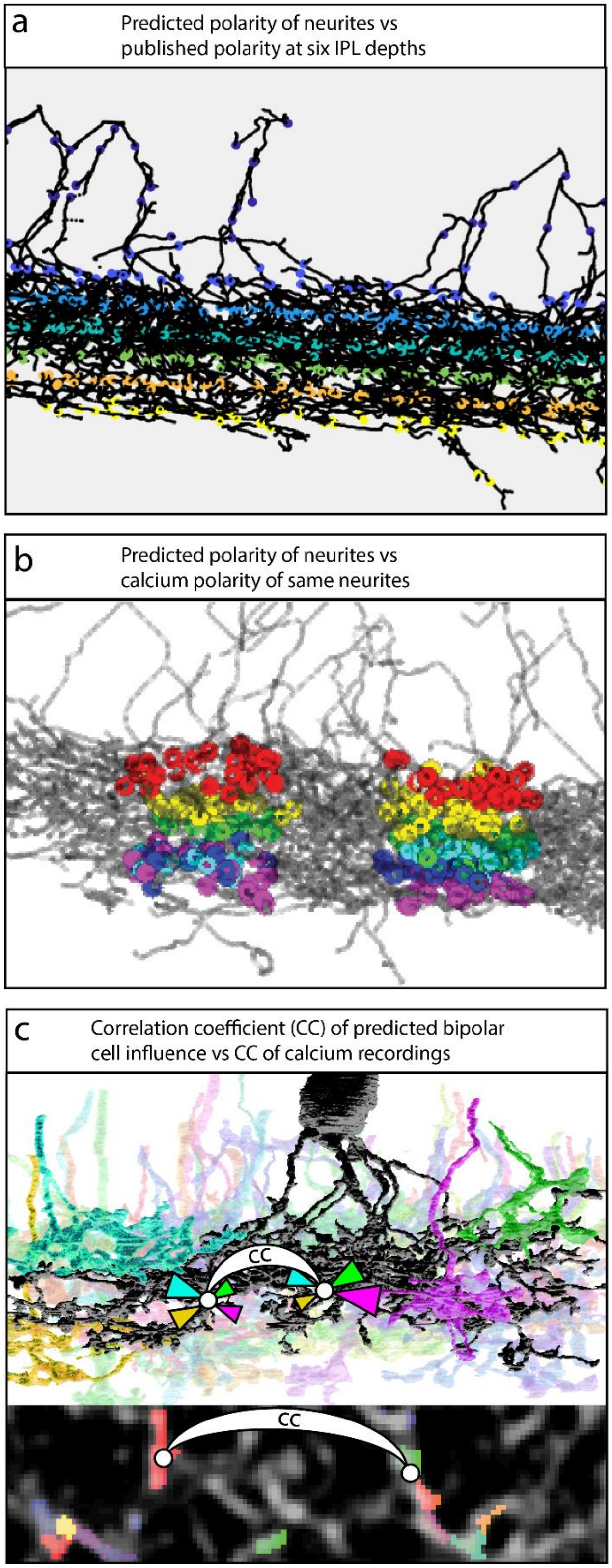

Supplementary Fig 6: Illustration of comparisons made for estimating length constant of calcium spread. **a.** Locations in EM reconstruction of VG3 plexus that have been matched to locations in functional calcium recordings. Prediction of ON/OFF polarity based on anatomical proximity to ON and OFF bipolar cell inputs is compared to ON/OFF polarity based on calcium responses to ON and OFF stimuli. Dot colors indicates functional imaging plane. **b.** EM predictions of ON/OFF polarity were compared to previously published reports of the shift of VG3 neurite polarity across IPL depth. Skeleton nodes at IPL depths matching previous reports of VG3 recordings were assigned the corresponding polarity for comparison. **c.** Anatomical and functional correlation coefficients were assigned for each light/EM correspondence point. Anatomical correlation coefficients (top) are calculated by creating a vector representing the predicted influence of bipolar cells and calculating the correlation coefficient between all pairs of correspondence points. Functional correlation coefficients (cc, bottom) were calculated by comparing the estimated calcium levels outside of the stimulus windows.

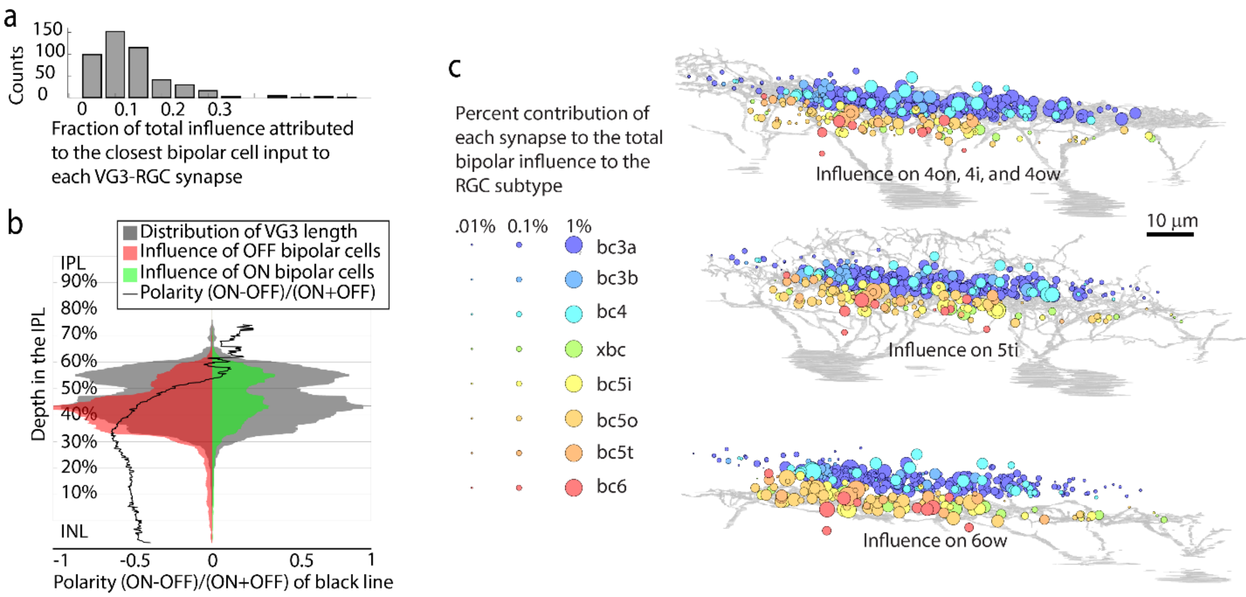

Supplementary Fig. 7: Bipolar influence on the VG3 plexus. **a,** Using a length constant of 16 μm, we calculated how much of the bipolar cell influence onto each VG3 to RGC synapse came from the nearest bipolar to VG3 synapse. For most VG3 to RGC synapses, the nearest bipolar cell input contributed less than 20% of the total bipolar cell influence. **b,** We calculated the total ON (green) and OFF (red) influence bipolar cells deliver across the depth of the VG3 plexus. The depth distribution of VG3 arbor length is shown in gray. Predicted polarity is shown in the black trace. **c,** Distribution of the bipolar cell synapses predicted to influence different RGC types through the VG3 plexus. Monostratified OFF cells (4on, 4i, 4ow) are grouped together in the top rendering. Each circle indicates the position of a bipolar cell to VG3 synapse. The color of the synapse indicates the type of bipolar cell. The size of the circle indicates the fraction of the total bipolar cell influence on the RGC type that can be attributed to that bipolar cell to the VG3 synapse.
