# Supplementary material for: Subcellular pathways through VGluT3-expressing mouse amacrine cells provide locally tuned object-motion-selective signals in the retina
Source: Nat Commun. 2024 Apr 5;15:2965. doi: 10.1038/s41467-024-46996-0 (PMC10997783; doi:10.1038/s41467-024-46996-0)
Supplement: Supplementary file 2 — Description of Additional Supplementary Files [file 41467_2024_46996_MOESM2_ESM.pdf]

Description of Additional Supplementary Files

Supplementary Movie 1: Rendering of reconstructed VG3 amacrine cells (red), bipolar cells (green), and RGCs (blue). Scalebar is 10 [illegible]m.
