# Supplementary material for: Subcellular pathways through VGluT3-expressing mouse amacrine cells provide locally tuned object-motion-selective signals in the retina
Source: Nat Commun. 2024 Apr 5;15:2965. doi: 10.1038/s41467-024-46996-0 (PMC10997783; doi:10.1038/s41467-024-46996-0)
Supplement: Supplementary file 5 — Reporting Summary [file 41467_2024_46996_MOESM5_ESM.pdf]

nature portfolio

Corresponding author(s): Josh Morgan, Daniel Kerschensteiner

Last updated by author(s): Feb 20, 2024

Reporting Summary

Nature Portfolio wishes to improve the reproducibility of the work that we publish. This form provides structure for consistency and transparency in reporting. For further information on Nature Portfolio policies, see our Editorial Policies and the Editorial Policy Checklist.

Statistics

For all statistical analyses, confirm that the following items are present in the figure legend, table legend, main text, or Methods section.

n/a	Confirmed	
☐	☒	The exact sample size (*n*) for each experimental group/condition, given as a discrete number and unit of measurement
☐	☒	A statement on whether measurements were taken from distinct samples or whether the same sample was measured repeatedly
☒	☐	The statistical test(s) used AND whether they are one- or two-sided<br>*Only common tests should be described solely by name; describe more complex techniques in the Methods section.*
☒	☐	A description of all covariates tested
☒	☐	A description of any assumptions or corrections, such as tests of normality and adjustment for multiple comparisons
☐	☒	A full description of the statistical parameters including central tendency (e.g. means) or other basic estimates (e.g. regression coefficient) AND variation (e.g. standard deviation) or associated estimates of uncertainty (e.g. confidence intervals)
☒	☐	For null hypothesis testing, the test statistic (e.g. *F*, *t*, *r*) with confidence intervals, effect sizes, degrees of freedom and *P* value noted<br>*Give P values as exact values whenever suitable.*
☒	☐	For Bayesian analysis, information on the choice of priors and Markov chain Monte Carlo settings
☒	☐	For hierarchical and complex designs, identification of the appropriate level for tests and full reporting of outcomes
☐	☒	Estimates of effect sizes (e.g. Cohen's *d*, Pearson's *r*), indicating how they were calculated

Our web collection on statistics for biologists contains articles on many of the points above.

Software and code

Policy information about availability of computer code

Data collection	Data was collected using the custom Matlab (Mathworks, v2015 32bit) software package Wafermapper (v2.01, https://github.com/MorganLabShare/VG3_2023 , DOI 10.5281/zenodo.10685346).
Data analysis	Data was analyzed using the following software. Tracing was performed manually using VAST (v1.2.1.33x, https://lichtman.rc.fas.harvard.edu/vast/). The custom Matlab (Mathworks, v2023a) code CellNav (v0.81, vVG3Pub2023, https://github.com/MorganLabShare/VG3_2023, DOI 10.5281/zenodo.10685346)) was used to analyze segmentations and compare anatomical models to physiological results. NEURON (v8.1, https://www.neuron. https://github.com/neuronsimulator/nrn.edu/neuron/) modeling was performed using the T2N interface (https://www.treestoolbox.org/T2N.html) for the Matlab (Mathworks) TREES (v1.15) module.

For manuscripts utilizing custom algorithms or software that are central to the research but not yet described in published literature, software must be made available to editors and reviewers. We strongly encourage code deposition in a community repository (e.g. GitHub). See the Nature Portfolio guidelines for submitting code & software for further information.

Data

Policy information about availability of data

All manuscripts must include a data availability statement. This statement should provide the following information, where applicable:

- Accession codes, unique identifiers, or web links for publicly available datasets
- A description of any restrictions on data availability
- For clinical datasets or third party data, please ensure that the statement adheres to our policy

All data will be made available upon request (raw data is several terabytes). Examples of the data are available at https://sites.wustl.edu/morganlab/vg3-data-code-and-more/.

Research involving human participants, their data, or biological material

Policy information about studies with human participants or human data. See also policy information about sex, gender (identity/presentation), and sexual orientation and race, ethnicity and racism.

Reporting on sex and gender	N/A
Reporting on race, ethnicity, or other socially relevant groupings	N/A
Population characteristics	N/A
Recruitment	N/A
Ethics oversight	N/A

Note that full information on the approval of the study protocol must also be provided in the manuscript.

Field-specific reporting

Please select the one below that is the best fit for your research. If you are not sure, read the appropriate sections before making your selection.

☒ Life sciences ☐ Behavioural & social sciences ☐ Ecological, evolutionary & environmental sciences

For a reference copy of the document with all sections, see nature.com/documents/nr-reporting-summary-flat.pdf

Life sciences study design

All studies must disclose on these points even when the disclosure is negative.

Sample size	Sample size was determined by the limits of electron micrographic circuit reconstruction. Data was collected from the retina of a single mouse. Statistical inference measures such as standard error were calculated using the number of cells as the n. This use of cell number as sample size was described within the results section of the text to make it clear that our results demonstrate patterns and specificity that the system is capable of but do not describe the potential variability between mice. Confidence intervals for effect sizes are provided whenever possible.
Data exclusions	None
Replication	The time required to reproduce the data collection and analysis presented here makes direct replication impractical. Consistency of results with previous literature is discussed.
Randomization	N/A
Blinding	Cell type assignments were performed by humans as described. Synapse counts were performed prior to cell type assignment.

Reporting for specific materials, systems and methods

We require information from authors about some types of materials, experimental systems and methods used in many studies. Here, indicate whether each material, system or method listed is relevant to your study. If you are not sure if a list item applies to your research, read the appropriate section before selecting a response.

Materials & experimental systems

n/a	Involved in the study
☒	☐ Antibodies
☒	☐ Eukaryotic cell lines
☒	☐ Palaeontology and archaeology
☐	☒ Animals and other organisms
☒	☐ Clinical data
☒	☐ Dual use research of concern
☒	☐ Plants

Methods

n/a	Involved in the study
☒	☐ ChIP-seq
☒	☐ Flow cytometry
☒	☐ MRI-based neuroimaging

Animals and other research organisms

Policy information about studies involving animals; ARRIVE guidelines recommended for reporting animal research, and Sex and Gender in Research

Laboratory animals	Tissue was collected from the offspring of a GCaMP6f mice (Ai148, JAX #030328) crossed to VGLUT3-Cre (Slc17a8-IRES2-Cre-D, Jax # 028534). The mouse was 67 days old
Wild animals	No wild animals were used in this study.
Reporting on sex	Sex was not considered in this analysis. The study is based on a reconstruction of a single mouse retina. We expect that any sex based variability in retina organization would be smaller than the sensitivity of our sampling.
Field-collected samples	No field collected animals were used in this study.
Ethics oversight	All experiments were approved by Washington University IACUC.

Note that full information on the approval of the study protocol must also be provided in the manuscript.

Plants

Seed stocks	n\a
Novel plant genotypes	n\a
Authentication	n\a
